# Throwing light on night blindness associated with rare manifestation of vitamin A deficiency

**DOI:** 10.22336/rjo.2024.78

**Published:** 2024

**Authors:** Avadhesh Oli, Sujata Upadhyay, Agrima Bhatia, Bhavaraj Veerabhadhra Rao

**Affiliations:** 1 Department of Ophthalmology, Command Hospital (Air Force) Bangalore, India; 2 Military Hospital Leh, India; 35 Air Force Hospital, Jorhat, Assam, India

**Keywords:** Mizuo phenomenon, Oguchi disease, multimodal imaging, Vitamin A deficiency, modified patch test

## Abstract

**Objective:**

This case report describes a rare presentation of the Mizuo phenomenon in a vitamin A deficiency-associated patient.

**Methods:**

A 38-year-old male presented with nyctalopia for 10 years and a fresh onset of dry eyes. He was a known case of alcoholic liver disease with cirrhosis and Oguchi disease. Anterior segment evaluation revealed moderate dry eyes, and fundus examination revealed metallic sheen OU, the latter extinguished after dark adaptation via a modified patch test suggestive of the Mizuo Nakamura phenomenon. Multimodal imaging modalities like SD-OCT, short-wave autofluorescence, and electrophysiological tests were performed. Findings suggested a diagnosis other than Oguchi’s disease. The gene panel comprising SAG and GRK1 mutations was negative for Oguchi. Serum vitamin A levels were significantly low (0.03 mg/l). The patient was started on topical lubricants and oral vitamin A supplements and counseled to abstain from alcohol.

**Results:**

SD-OCT demonstrated thinning of the ellipsoid region with multiple hyperreflective excrescences in outer retinal layers colocalized with hyperautofluorescent flecks on short-wave autofluorescence. ERG was normal bilaterally. An electrooculogram showed a significantly decreased Arden ratio of OU. After controlled oral Vitamin A supplementation, the patient’s serum Vitamin A levels remained low (0.06 mg/l).

**Conclusion:**

This case is rare and likely the first reported presentation of the Mizuo phenomenon in a vitamin A deficiency-associated patient with normal ERG and abnormal EOG. Increasing choice for weight loss diet and bariatric surgeries Vitamin A deficiency is also expected to affect developed countries. Modified patch tests make diagnosis easier and earlier. More extensive studies are needed to correlate levels of Vitamin A with the natural course of the Mizuo phenomenon and substantiate multimodal imaging findings.

## Introduction

Vitamin A deficiency is rare in developed and nutrient-rich countries but is still prevalent in developing countries. Vitamin A deficiency-associated ocular symptoms have been shown to occur at concentrations of less than ten micrograms/dL [1]. Besides nutritional deficit, pancreatic, liver, and intestinal pathology are the leading causes of vitamin A deficiency. Chronic liver disease of any type has been associated with vitamin A deficiency. It is an acquired form of rod photoreceptor disease leading to night blindness. Vitamin A deficiency can lead to night blindness due to poor visual pigment regeneration in retinal rods [2]. Many inherited retinal pathologies manifest as night blindness (spectrum of congenital stationary night blindness). One is Oguchi disease with characteristic retinal findings, called the Mizou-Nakamura phenomenon, in which golden-yellow fundus discoloration disappears after prolonged dark adaptation.

We present a case of Severe Vitamin A deficiency in a patient with alcoholic liver cirrhosis with portal hypertension who had presented with nyctalopia for the past 10 years and dry eye symptoms for the past few months.

## Case report

**Fig. 1:**
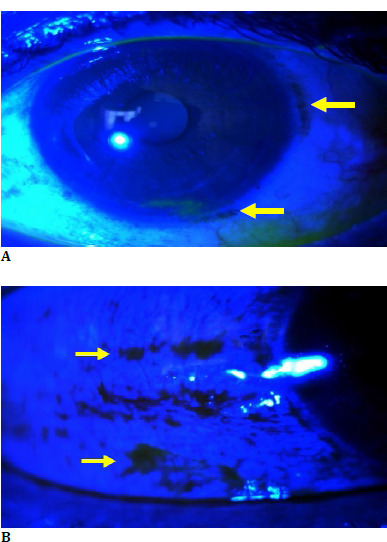
**A, B**. Anterior segment evaluation suggestive of conjunctival xerosis and moderate dry eyes

A 38-year-old male patient presented to us with complaints of nyctalopia for the past 10 years and foreign body sensation in both eyes for the past few months. There was no family history of consanguinity or any ocular disorder. He was a known case of alcoholic liver disease with cirrhosis and Oguchi disease. The patient’s best-corrected visual acuity was 20/40 in the Right Eye and 20/20 in the Left eye; color vision, as determined with pseudoisochromatic Ishihara color plates, was expected in each eye. Anterior segment evaluation suggested moderate dry eyes with a tear break time of 5 seconds OU (**Fig. 1 A, B**).

**Fig. 2:**
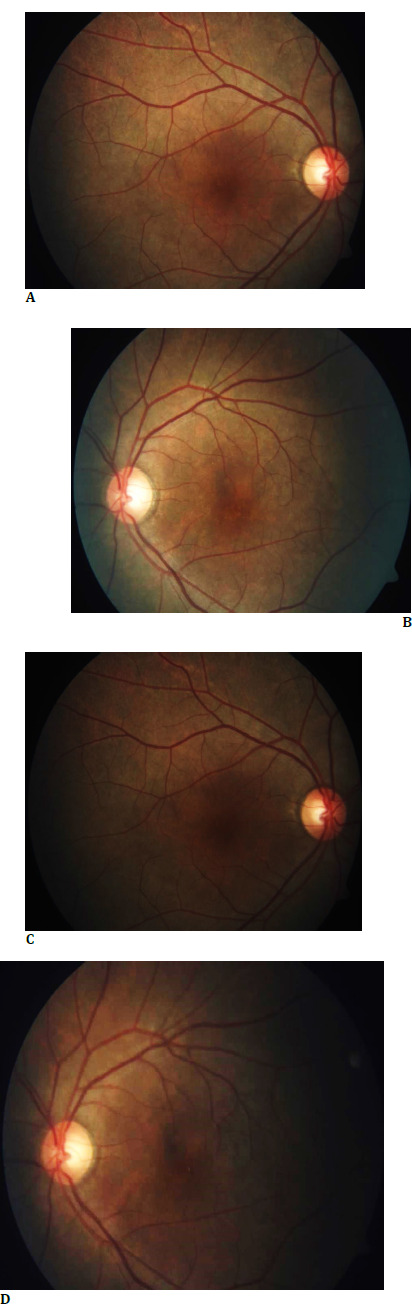
**A, B**. Fundus examination revealed metallic sheen in both eyes; **C, D**. Extinguished metallic sheen in both eyes post-dark adaptation of 1 hour

**Fig. 3:**
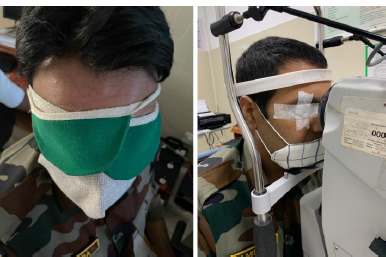
Modified dark adaptation (Patch) test using a cloth patch

Fundus examination revealed metallic sheen in both eyes (**Fig. 2 A, B**), which disappeared after dark adaptation for 1 hour (**Fig. 2 C, D**). Due to time and space constraints, we chose a modified dark adaptation test wherein instead of making the patient sit in a dark room for 3-4 hours, we patched both his eyes for 1 hour with a cloth patch to obstruct the light completely (**Fig. 3**).

**Fig. 4:**
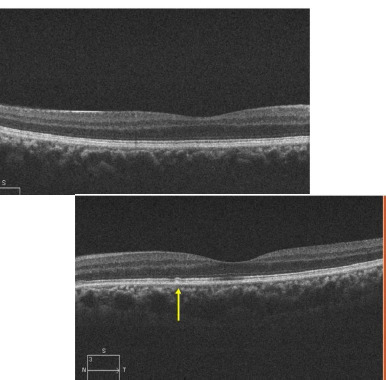
Spectral Domain Optical Coherence tomography cross sections demonstrated thinning of the ellipsoid region with multiple hyperreflective excrescences in the outer retinal layers

**Fig. 5:**
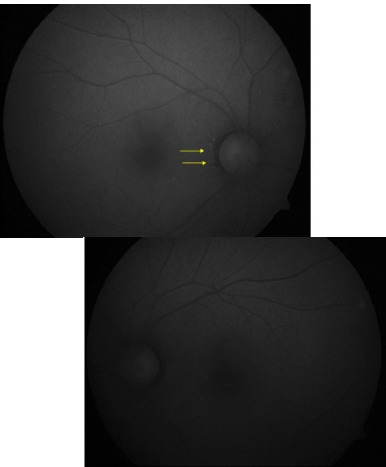
Hyperautofluorescent flecks on short-wave autofluorescence imaging

The golden sheen of the fundus, which was extinguished after dark adaptation, was suggestive of the Mizou Nakamura phenomena. Interestingly, the patient had been diagnosed with Oguchi disease ten years back at another institution, underscoring the importance of re-evaluation in cases with overlapping clinical features. We, however, chose to perform a meticulous examination protocol as the symptoms of nyctalopia were persistent with the fresh onset of features of dry eyes. He showed increased dark and light adaptation time (approx. 45 minutes). Multimodal imaging modalities like Spectral Domain Optical Coherence tomography cross sections demonstrated thinning of the ellipsoid region with multiple hyperreflective excrescences in the outer retinal layers (**Fig. 4**) colocalized with hyperautofluorescent flecks on short-wave autofluorescence imaging (**Fig. 5**).

**Fig. 6:**
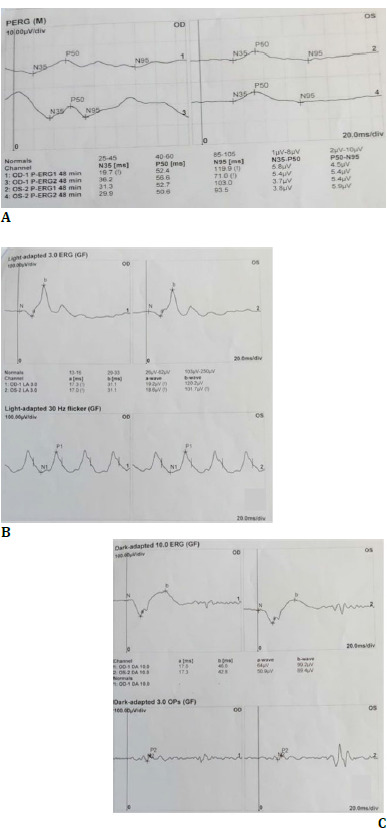
**A-C**. Pattern, Scotopic, and Photopic ERG were regular bilaterally

**Fig. 7:**
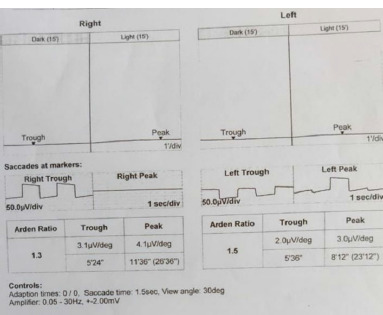
Electrooculogram showed a significantly decreased Arden ratio in both eyes

The electrophysiological tests performed according to ISCEV standards were utilized to navigate the diagnosis objectively. Pattern, scotopic, and photopic ERG were normal bilaterally (**Fig. 6 A-C**). Electrooculogram showed a significantly decreased Arden ratio (OD 1.3 and OS 1.5) (**Fig. 7**). This raised suspicion of an underlying additional retinal pathology or a diagnosis other than Oguchi disease. To aid in unraveling this perplexity, a gene panel comprising SAG and GRK1 mutations was conducted, which yielded negative results for Oguchi. Serum vitamin A levels, however, were detected to be significantly low (0.03 mg/l).

The significantly low vitamin A levels, slowly progressive night blindness, and gradually worsening dry eye symptoms favored Vitamin A deficiency-associated retinal pathology with associated Mizou Nakamura phenomena. The patient was started on topical preservative-free lubricants and oral vitamin A supplements and counseled to abstain from alcohol. The Vitamin A supplementation was limited and gradual (20000 IU/day) as we intended not to aggravate the liver dysfunction. After the controlled oral supplementation of Vitamin A, the patient’s serum Vitamin A levels persisted to be low (0.06mg/l), and there has been no relief in nyctalopia, probably due to persistent low vitamin A levels.

## Discussion

Vitamin A deficiency associated with xerophthalmic fundus is a well-recognized pathology. Our patient's vitamin A level was 1000 times below the minimum reference level of 0.3-1.2 mg/L.

The functional retinal changes precede structural changes. Nyctalopia is the earliest manifestation of vitamin A-associated xerophthalmic fundus. It is also associated with depressed ERG, which depends on the age of manifestation [3]. Various diseases associated with the metallic sheen of the fundus are Oguchi disease, RS1 mutation, RDH5 or RLBP1-related disease [4], and *RGS9/R9AP* mutation [5]. Two genes acting in sequence to cease the phototransduction cascade and reported mutations that cause Oguchi disease are the *SAG(Arrestin)* and *GRK1(Rhodopsin Kinase)* [6]. The typical metallic sheen of the fundus with normal cone function, delayed rod ERG dark adaptation, and marked rod desensitization to a bright flash is distinctive for Oguchi disease [7]. The color change is thought to be caused by an excess of extracellular potassium in the retina due to a decreased potassium scavenging capacity of retinal Müller cells [8]. A normal ERG and an adverse genetic profile ruled out Oguchi disease or retinal gene disorders in this patient. However, there is a documented case of decreased Arden ratio in Vitamin A deficiency, as seen in this case [9]. The literature review reveals varied structural manifestations like macular pigmentation and white dots on the fundus. Still, no metallic sheen (Mizou phenomena) has been reported in vitamin A deficiency. Hypothetically, the hyperreflective dots in the outer retina correspond to both loss and accrual of photoreceptor segments secondary to decreased phagocytosis by RPE cells in vitamin A deficiency [10]. Clinically, they correspond to the retinal flecks.

## Conclusion

Hence, this case is a rare and likely the first reported presentation of the Mizuo phenomenon in a vitamin A deficiency-associated patient with normal ERG and abnormal EOG. As the patient was previously diagnosed with Oguchi disease, it underscores the importance of re-evaluation in cases with overlapping clinical features. Increasing choice for weight loss diet and bariatric surgeries Vitamin A deficiency is also expected to affect developed countries. Modified patch tests might aid in more accessible and earlier diagnosis. More extensive studies are needed to correlate levels of Vitamin A with the natural course of the Mizuo phenomenon and substantiate multimodal imaging findings.

## References

[1] Miller M, Humphrey J, Johnson E, Marinda E, Brookmeyer R, Katz J (2002). Why do children become vitamin A deficient?. J Nutr.

[2] Gilbert C (2013). The eye signs of vitamin A deficiency. Community Eye Health.

[3] Dhanda RP (1955). Electroretinography in Night Blindness and Other Vitamin-A Deficiencies. Archives of Ophthalmology.

[4] Robson AG, Mengher LS, Tan MH, Moore AT (2009). An unusual fundus phenotype of inner retinal sheen in X-linked retinoschisis. Eye (Lond).

[5] Nishiguchi KM, Sandberg MA, Kooijman AC, Martemyanov KA, Pott JW, Hagstrom SA (2004). Defects in RGS9 or its anchor protein R9AP in patients with slow photoreceptor deactivation. Nature.

[6] Fujinami K, Tsunoda K, Nakamura M, Oguchi Y, Miyake Y (2011). Oguchi Disease With Unusual Findings Associated With a Heterozygous Mutation in the SAG Gene. Arch Ophthalmol.

[7] Rishi P, Rishi E, Abraham S (2018). Oguchi’s disease with Mizuo-Nakamura phenomenon in a seven-year-old boy. GMS Ophthalmol Cases.

[8] Bergsma DR, Chen CJ (1997). The Mizuo phenomenon in Oguchi disease. Arch Ophthalmol.

[9] Leguire LE, Pappa KS, McGregor ML, Rogers GL, Bremer DL (1992). Electro-oculogram in vitamin A deficiency associated with cystic fibrosis. Short communication. Ophthalmic Paediatr Genet..

[10] Berkenstock MK, Castoro CJ, Carey AR (2020). Outer retina changes on optical coherence tomography in vitamin A deficiency. Int J Retina Vitreous.

